# Maternal smoking during pregnancy and academic achievement of offspring over time: A registry data-based cohort study

**DOI:** 10.1016/j.ypmed.2018.05.017

**Published:** 2018-08

**Authors:** Alfgeir L. Kristjansson, Sabena Thomas, Christa L. Lilly, Ingibjorg E. Thorisdottir, John P. Allegrante, Inga Dora Sigfusdottir

**Affiliations:** a Department of Social and Behavioral Sciences, School of Public Health, West Virginia University, Morgantown, WV 26505, USA; b Icelandic Center for Social Research and Analysis, Reykjavik University, 101 Reykjavik, Iceland; c Department of Biostatistics, School of Public Health, West Virginia University, Morgantown, WV, USA; d Department of Psychology, Reykjavik University, 101 Reykjavik, Iceland; e Department of Health and Behavior Studies, Teachers College, Columbia University, New York 10027, NY, USA; f Mailman School of Public Health, Columbia University, New York 10032, NY, USA

**Keywords:** Maternal smoking during pregnancy, Prenatal tobacco exposure, LICEFOURSE, Iceland, Cohort study, Academic achievement

## Abstract

Few studies have assessed the cumulative impact of maternal smoking during pregnancy (MSDP) on scholastic outcomes over time. We examined the relations between MSDP and academic achievement in the 4th, 7th and 10th grades using registry data collected at birth, during the neonatal period, and at each grade level from the 2000, LIFECOURSE study birth cohort in Reykjavik, Iceland (*N* = 1151, girls = 49.3%). Latent growth modeling showed that MSDP influenced Icelandic achievement scores, standardized to a range from 0 to 60, at baseline (β = −0.04), and over time (β = −0.05). Likewise, MSDP was negatively associated with standardized mathematics scores at baseline (ß = −0.09) and continued to exert a negative impact on mathematics scores over time (ß = −0.08) after controlling for gender, income, cohabitation, and baseline mathematics and Icelandic achievement scores. Results provide evidence of the persistent negative impact of MSDP on academic achievement in offspring. Findings support the proposition that children whose mothers smoke during the first trimester of pregnancy are, on average, at greater risk for poor scholastic outcomes over time than children whose mothers do not smoke during their first trimester. To our knowledge, this is the first study using a longitudinal cohort design to assess whether the impacts of maternal smoking during pregnancy may persist over time. This study contributes to the current state of knowledge by providing an assessment that focuses on the impact of smoking during pregnancy on academic achievement from childhood into early adolescence.

## Highlights

• Maternal smoking during pregnancy (MSDP) negatively impacts child development.
• MSDP negatively influences mathematics achievement over time.
• MSDP negatively influences language achievement over time.

## 1 Introduction

Maternal smoking during pregnancy (MSDP) has been shown to be negatively related to offspring's physical development, both in fetal and newborn stages, with corresponding impact reaching into infancy, adolescence and even young adulthood (Clifford et al., 2012; Gilman et al., 2008; Inamdar et al., 2015; Ion et al., 2015; Ko et al., 2014; Lanting et al., 2009; Polanska et al., 2015; Roelands et al., 2009; Rogers, 2009). Among the antepartum and postpartum developmental complications associated with MSDP are spontaneous abortion (Pineles et al., 2014), ectopic pregnancy (Roelands et al., 2009), pre-eclampsia (Roelands et al., 2009; Wikström et al., 2010), placental rupture (Jauniaux and Burton, 2007), fetal growth restriction, pre-term birth, and low birth weight (Ko et al., 2014). MSDP has also been associated with neurological and cognitive dysfunctions such as impediments to brain development among infants (Wehby et al., 2011), poor visual-motor integration and verbal competence in young children (Heinonen et al., 2011), lower IQ, reduced scholastic achievement, and intellectual impairments in grade school children (Alloway and Alloway, 2010; Cornelius et al., 2012; Duckworth and Seligman, 2005; Kafouri et al., 2009; Pineles et al., 2014; Braun et al., 2009; Gilman et al., 2008; Huijbregts et al., 2006), and behavioral problems such as conduct disorders and attention deficit hyperactivity disorders (ADHD) in children aged from 6 to 16 years (Gaysina et al., 2013; Langley et al., 2007).

Several studies have identified a reduction in scholastic achievement among the offspring of mothers who smoked tobacco during pregnancy (Lambe et al., 2006; Martin et al., 2006; O'Callaghan et al., 2010). For instance, Lambe et al. (2006) examined over 400,000 15-year-old Swedish students and their school performance measured by grade-point summary scores in 16 subjects in relation to their prenatal cigarette smoke exposure. The results showed a deficit among those who were exposed to prenatal cigarette smoke. A Finnish study assessed the mean academic grades for all subjects among 12-year-old children, and found a reduction in scholastic achievement among children of smokers compared to non-smokers (Martin et al., 2006). Similar results were also reported in an Australian prospective study of over 7000 mothers and their children (O'Callaghan et al., 2010). Conversely, data from the US National Collaborative Project, which included a sample of ~50,000 participants, found no association between MSDP and scholastic achievement measured by standardized scores from reading, spelling and arithmetic (Gilman et al., 2008). This result was consistent with a Swedish quasi-experimental study using a sibling-comparison approach among 15-year-old students; there were no differences in school grades and math proficiency among the exposed and unexposed siblings (D'Onofrio et al., 2010).

In addition to the inconsistency in current knowledge regarding the relations between MSDP and academic outcomes in the offspring, few studies have sought to assess the impact of MSDP on scholastic outcomes at multiple time points, despite the need and calls for such studies (Martin et al., 2006). The few studies that have included an assessment of academic outcomes at more than one time period have not employed statistical models that account for the linked time structure in the data and hence the potential for a cumulative effect over time. For example, Gilman et al. (2008) assessed the relations between prenatal smoke exposure and academic achievement in 4- and 7-year-old children respectively, but found no association after controlling for potential confounders, and Kristjansson et al. (2017) found negative relations between MSDP and academic achievement in the offspring at ages 10, 13 and 15. Thus, in order to understand the cumulative, long-term impact of MSDP on academic achievement, studies need to employ consistent measures that capture academic achievement over several developmental periods, and analytic methods that tests them simultaneously in one coherent model.

Another related problem with previous assessments has been the variation in covariates studied. These include both birth-related factors such as birth weight and familial characteristics that may contribute to the relationship between MSDP and cognitive outcomes (e.g., Gilman et al., 2008; Lawlor et al., 2006), while other studies have indicated that familial factors such as mother's education are more likely than birth related measures to explain cognitive outcomes (Batty et al., 2006; Breslau et al., 2005; Clifford et al., 2012; Wehby et al., 2011). At the same time, a few studies have included an extensive range of birth-related outcomes, but have omitted familial and social factors at time of assessment (Lambe et al., 2006), and others have included social and familial characteristics at the time of assessment but have failed to include birth-related factors (O'Callaghan et al., 2010). Given the relative importance of these two sets of covariates and appeals for more consistency in the selection of covariates (Clifford et al., 2012; Inamdar et al., 2015; Ion et al., 2015; Ko et al., 2014; Lanting et al., 2009; Roelands et al., 2009), studies that incorporate both while simultaneously controlling for social and familial factors at different assessment periods could better illuminate the relationship between MSDP and academic achievement.

The aim of this study was to improve the current understanding of the potential long-term impact of MSDP on academic achievement. We examined the impact of MSDP on standardized academic scores at the age of 10–11 (4th grade), 13–14 (7th grade), and 15–16 (10th grade), with data collected at birth, during the neonatal period, and at each grade level. We hypothesize that maternal smoking would lead to both lower baseline achievement scores, as well as cumulative lower scores over time.

## 2 Methods

### 2.1 Sample and participants

This report is based on data from the LIFECOURSE study of risk and protective factors being conducted by the Centre for Social Research and Analysis (ICSRA) at Reykjavik University in Iceland. LIFECOURSE is a developmental cohort study that covers the early lifespan of a birth cohort of children from before birth to the age of 15/16. The theoretical framework for the study has been described elsewhere (Sigfusdottir et al., 2017). The study sampling frame consists of all children born, and residing in, Reykjavik, Iceland, in the year 2000 (*N* = 1151, girls = 49.3%). Study material for the investigation comprised a combination of official registry data from several national data banks assembled from 2014 to 2016. For the purpose of this analysis, we used retrospective registry data from the following sources: i) The National Birth Registry at the Landspitali University Hospital, ii) Antenatal records from the Mother care registry at the Primary Health Care Clinics, both overseen by the Icelandic Directorate for Health which oversees the entire health registry system in Iceland, iii) the Educational Testing Institute (ETI) overseen by the Ministry of Education, Science, and Culture, and iv) the Statistical Bureau of Iceland. The study was reviewed and approved by the National Bioethics Committee of Iceland (equivalent to a national IRB) and the study has been registered and acknowledged by the Personal Protection Authority.

### 2.2 Measures

#### 2.2.1 Achievement

Achievement score data in mathematics and the Icelandic language in 4th (aged 10–11), 7th (age 13–14), and 10th (age 15–16) grades came from the ETI database. Grading in Iceland is given on a numerical scale from 0 to 10 with 5 and above as “passed” and 7.25 and above regarded as first-class grade. For the purpose of this assessment, the ETI delivered standardized grades on a scale from 0 to 60 for each subject, with mean scores fixed at 30, and a combined scale for the two subject ranging from 0 to 120.

#### 2.2.2 Annual household income

Data on the total income for the household were available by year, and calculated in the survey as the additive combination of income from employment, capital, and any other income revenue. Explorative analysis of all year's data revealed that the final available year of income, 2013, had the strongest statistical relationship to the achievement variables. This variable, ranging from 0 to 172 million ISK, was further split into deciles, with 10% in each group, in order to account for excess 0’s and very strong positive skew.

#### 2.2.3 Maternal smoking

Maternal smoking during pregnancy was assessed during the first antenatal visit which usually takes place towards the end of the first trimester in Iceland. Expecting mothers were asked whether they currently smoke tobacco or if they did so before knowing about becoming pregnant. Smoking status was initially coded as (0) no, (1) yes, sometime during pregnancy, and (2) yes, before knowing about pregnancy. Previous work (Kristjansson et al., 2017) demonstrated that those endorsed the final option (10% of the population) were more similar to those who said no than to regular cigarette smokers. Thus, we recoded this variable into (1) yes, sometime during pregnancy and (0) no smoking or smoking prior to knowing about pregnancy.

#### 2.2.4 Cohabitation at birth

Maternal cohabitation with the father at time of birth data came from the birth registry database, and was recoded into (1) yes, (0) no.

#### 2.2.5 Gender of child

Sex at birth data came from the birth registry database, and was recoded into (1) male, (0) female.

### 2.3 Procedure

Contact information for the sample was acquired through the national Statistical Bureau and sister agencies. A non-traceable research identification number was created for each participant and flash drives with this information delivered to local personnel at each site with the proper authority to handle the sensitive and personal information. The data collected were then prepared and transferred to files at each site during the years 2014–2016 using the research ID number to identify participants while removing all personal information upon delivery of the data files to the research team. Available data for each variable in the registry material ranged from 980 to 1149 or 85.1% to 99.8% of the study sample. A key that links individual names and contact information to research IDs is maintained by a third party at the Primary Health Care Clinics and is not accessible to the research team.

### 2.4 Statistical analysis

All analyses were conducted in SAS v. 9.4 (SAS, 2017). Descriptive statistics were generated for all variables included in the analysis, i.e., frequency and valid percent reporting for categorical or binary variables, and mean, standard deviation, and minimum and maximum values for continuous variables. Pearson, Point-Biserial, and Kendall's tau correlations were calculated for all study variables as appropriate for the variable type. Additional independent sample *t*-tests, with alpha set to 0.05, were included for testing differences between each achievement measure and maternal smoking status.

To test the key research question concerning the impact of maternal smoking status on child achievement scores over time, we followed a latent growth model (LGM) analytic approach that utilized structural equation modeling allowing repeated measures over time via latent intercepts and slopes. This analytic technique has advantages over more traditional methods of repeated measures analysis by addressing individual differences in growth trajectories in terms of latent variables (i.e., allowing latent variables to represent intercepts and slopes over time; Curran and Muthen, 1999; McArdle, 1988). In this model, the intercept parameter estimate is interpreted as the predicted growth curve when the set of predictors are all equal to 0 (i.e., at baseline), and the slope parameter estimate is interpreted as the predicted growth curve for each unit change in time.

A series of models was conducted following the approach recommended by Tan et al. (2010) and modified as needed for updates in the SAS PROC CALIS procedure. This approach included maximum likelihood estimation, testing measurement components related to the repeated measures (such as linear and non-linear growth, auto regression and moving average, or ARMA), first on each individual set of achievement measures and then on the combination of achievement measures, including multivariate, associative, factor-of-curves, conditional factor-of-curves, and related ARMA factor-of-curves LGM.

Additional measurement testing included common slopes and intercepts across the measurement models and assessment of correlated errors. Models were compared with a variety of goodness of fit measures, including Akaike's Information Criteria (AIC) with preferred lowest across models, non-significant model chi-square (*P* > 0.05), Comparative Fit Index (CFI) > 0.95, and Root Mean Square Error of Approximation (RMSEA) < 0.05 or 90% confidence interval including 0.05. Once the best fitting measurement model was determined, structural components were included in the model as suggested by theoretical model appropriateness and statistical fit.

Additional testing was done by assessing a model without gender against; 1) a model with gender as a covariate and, 2) gender as a stratification variable for group-based differences. A group-based model, (gender stratification) produced the poorest fit. The best fitting models are presented both with and without gender as a covariate, while testing the nested model for chi-square differences where improved fit for a more complex model was determined as a significant (*P* < 0.05) difference test. Parameter estimates, including standardized and unstandardized beta coefficients with t-values, are presented for the structural models.

## 3 Results

### 3.1 Descriptive statistics

Table 1 presents correlations for all study variables. Around a quarter of the sample (19%) reported smoking during pregnancy, and yet significant negative correlations were found between maternal smoking and all study variables except for male gender. Independent sample *t*-tests showed significant (all *p* < 0.01) differences in mathematics achievement, with those who reported maternal smoking having lower means compared with no maternal smoking in 4th grade: 27.69 (9.81) v. 31.52 (10.37), 7th grade: 27.69 (9.91) v. 31.66 (9.98), and 10th grade: 28.06 (10.52) v. 32.48 (10.11). Differences were also found, albeit not as strong, for Icelandic achievement scores, including 4th grade: 29.75 (10.16) v. 32.16 (10.30), *P* = 0.01, 7th grade: 28.99 (10.73) v. 31.90 (10.12), *P* = 0.01, and 10th grade: 29.03(10.28) v. 32.58 (10.57), *P* < 0.01.

**Table 1:** Correlations and descriptive statistics. The LIFECOURSE cohort study, Iceland.

Measure	1	2	3	4	5	6	7	8	9	10
1. Deciles, Total income in 2013	–									
2. Male	−0.015	–								
3. Maternal smoking	−0.226⁎⁎⁎	0.008	–							
4. Cohabitation at birth	0.269⁎⁎⁎	0.008	−0.201⁎⁎⁎	–						
5. Math, grade 4	0.201⁎⁎⁎	0.070⁎	−0.142⁎⁎⁎	0.057	–					
6. Math, grade 7	0.213⁎⁎⁎	−0.011	−0.152⁎⁎⁎	0.120⁎⁎	0.701⁎⁎⁎	–				
7. Math, grade 10	0.269⁎⁎⁎	−0.02	−0.166⁎⁎⁎	0.127⁎⁎⁎	0.617⁎⁎⁎	0.751⁎⁎⁎	–			
8. Icelandic, grade 4	0.172⁎⁎⁎	−0.134⁎⁎⁎	−0.089⁎⁎	0.061	0.622⁎⁎⁎	0.530⁎⁎⁎	0.512⁎⁎⁎	–		
9. Icelandic, grade 7	0.212⁎⁎⁎	−0.143⁎⁎⁎	−0.109⁎⁎	0.130⁎⁎⁎	0.598⁎⁎⁎	0.674⁎⁎⁎	0.618⁎⁎⁎	0.784⁎⁎⁎	–	
10. Icelandic, grade 10	0.242⁎⁎	−0.219⁎⁎	−0.131⁎⁎⁎	0.106⁎⁎	0.561⁎⁎⁎	0.651⁎⁎⁎	0.730⁎⁎⁎	0.710⁎⁎⁎	0.807⁎⁎⁎	–

Descriptive statistics										
N	1079	1149	1103	1149	1026	1043	997	1018	1059	1009
Mean or N	4.50	582	210	956	30.64	30.83	31.57	31.62	31.23	31.81
STD or %	2.87	50.65%	19.04%	83.20%	10.37	10.05	10.26	10.38	10.30	10.55
Minimum	0	–	–	–	0	0	0	0	0	0
Maximum	9	–	–	–	60	58	60	59	60	60

Correlations are Pearson for continuous variables, Point-Biserial for continuous with binary variables, and Kendall's tau for binary variables.

⁎⁎⁎ *p* < 0.0001.

⁎⁎ *p* < 0.001.

⁎ *p* < 0.05.

### 3.2 Latent growth models

The best fitting measurement model was an associative LGM with correlated errors between mathematics and Icelandic grades at 4th grade, at 7th grade, and at 10th grade. Best-fitting structural additions included allowing a cross-lagged structure between the intercepts on slopes, allowing correlations between disturbances for slopes and for intercepts, and covariates which selectively impacted the intercepts and slopes for mathematics and Icelandic achievement. Table 2 shows the model fit and comparisons for the final model excluding gender as a covariate, gender as a covariate, and stratified by gender. The chi-square difference test suggests both models fit well (*P* = 0.11) and thus including gender as a covariate does not hurt our model fit. Excellent model fit was obtained for the final model. Table 3 gives the parameter estimates for the model including gender as a covariate. Notably, maternal smoking is negatively associated with baseline math scores (ß = −0.09) after controlling for gender and income. There is moderate statistical evidence that it continues to negatively impact math achievement scores over time (β = −0.08) after controlling for gender, income, cohabitation, and baseline math and Icelandic achievement scores. Maternal smoking similarly although more weakly influences baseline Icelandic achievement scores (β = −0.04) and Icelandic achievement over time (β = −0.05).

**Table 2:** Model fit and comparisons.

Model	Number of parameters	Chi-square test of model fit	DF	*P*-value	AIC	RMSEA	90% CI RMSEA	CFI
No gender	42	12.13	12	0.444	96.03	0.002	0.00–0.04	1.000
Stratified by gender	42	172.38	66	<0.0001	256.38	0.064	0.05–0.08	0.973
Gender as a covariate	48	21.17	17	0.219	117.18	0.018	0.00–0.04	0.999
Models compared		Chi-square test of difference	DF	*P*-value				
No gender against covariate		9.04	5	0.11				

DF: Degrees of freedom.

AIC: Akaike's Information Criteria.

RMSEA: Room mean square error of approximation.

CFI: Comparative fit index.

**Table 3:** Structural parameter estimates for best-fitting model, gender included as covariate (*N* = 792).

Dependent variable	Independent variable	Unstandardized estimates	Standard error	Standardized	*t* value	R-square
Mathematics intercept		27.853	0.807		34.525⁎⁎⁎	0.067
	Deciles, Total income in 2013	0.673	0.125	0.214	5.392⁎⁎⁎	
	Male	1.303	0.688	0.074	1.894	
	Maternal smoking	−2.140	0.938	−0.090	−2.283⁎	
Mathematics slope		2.428	0.874		2.778⁎	0.136
	Math intercept	−0.141	0.043	−0.409	−3.314⁎⁎	
	Icelandic intercept	0.044	0.037	0.131	1.190	
	Deciles, Total income in 2013	0.129	0.055	0.119	2.353⁎	
	Male	−0.450	0.313	−0.073	−1.436	
	Maternal smoking	−0.660	0.392	−0.081	−1.686	
	Cohabitation	0.826	0.394	0.097	2.097⁎	
Icelandic intercept		30.590	0.811		37.741⁎⁎⁎	0.061
	Deciles, Total income in 2013	0.626	0.125	0.193	4.992⁎⁎⁎	
	Male	−2.516	0.692	−0.138	−3.638⁎⁎	
	Maternal smoking	−0.996	0.942	−0.041	−1.057	
Icelandic slope		1.392	0.850	–	1.637	0.226
	Icelandic intercept	−0.182	0.036	−0.629	−5.100⁎⁎⁎	
	Math intercept	0.143	0.036	0.482	4.011⁎⁎⁎	
	Deciles, Total income in 2013	0.079	0.049	0.085	1.613	
	Male	−1.383	0.279	−0.263	−4.950⁎⁎⁎	
	Maternal smoking	−0.374	0.348	−0.053	−1.075	
	Cohabitation	0.583	0.353	0.080	1.653	
Covariances	Maternal smoking and income	−0.201	0.038	−0.191	−5.285⁎⁎⁎	
	Cohabitation and income	0.267	0.037	0.263	7.154⁎⁎⁎	
	Cohabitation and maternal smoking	−0.028	0.005	−0.211	−5.797⁎⁎⁎	

⁎⁎⁎ *p* < 0.0001.

⁎⁎ *p* < 0.001.

⁎ *p* < 0.05.

## 4 Discussion

MSDP has significant negative, and educationally relevant, impact on baseline standing in mathematics and Icelandic achievement scores, and continues to exert negative impact on these scores over time. Bi-variate relationships revealed that male gender was not associated with MSDP; child gender may act as a moderator in the relationship between MSDP and academic achievement, we included a model with and without gender as a covariate. As expected, independent samples tests provided evidence that the offspring of those who reported maternal smoking during the first trimester of pregnancy had lower scores on average for both subjects (mathematics and Icelandic) at the 4th, 7th and 10th grades in comparison to those whose mothers did not smoke during the first trimester of pregnancy.

We extended our analytical approach with LGM, with and without gender as a mediator. The LGM models further demonstrated the negative impact of MSDP on academic performance for both mathematics and Icelandic achievement scores. More specifically, maternal smoking was predictive of a reduction in math scores at baseline and over time, irrespective of gender. Similar results were observed for Icelandic achievement scores at baseline and over time, although the magnitude of effects was not as large as mathematics scores. These results suggest that the negative effects of pre-natal exposure to maternal smoking may negatively affect the cognitive abilities of offspring and, perhaps more profoundly, that this negative effect may persist over time.

There are several plausible explanations for these findings. First, the carbon monoxide in cigarette smoke absorbed by mother and fetus creates high levels of carboxyhemoglobin; this reduces intrauterine oxygenated blood flow and may result in fetal hypoxia, intrauterine growth restriction and low birth weight (Liu et al., 2007; Mezzacappa et al., 2011; Rogers, 2009; Soothill et al., 1996). Second, nicotine has been identified as neuroteratogen that alters the neural development passageways of the brain and may reduce brain growth and head circumference (Dwyer et al., 2009; Pauly and Slotkin, 2008; Roza et al., 2007). Reduced head circumference in low birth weight infants is associated with reduced cognitive and neuropsychological abilities in school-age children (Lundberg et al., 2010). Pre-natal nicotine exposure has also been linked to post-natal neurobehavioral defects such as attention deficit-disorder, learning disabilities, hyperactivity and increased risk of behavioral problems (Lundberg et al., 2010; Pauly and Slotkin, 2008; Peterson et al., 2006; Rogers, 2009; Roza et al., 2007).

The evidence from this study is consistent with previous research, which has identified a reduction in scholastic achievement in the offspring exposed to maternal smoking during pregnancy (Lambe et al., 2006; Martin et al., 2006; O'Callaghan et al., 2010). Earlier studies examining the relationship between MSDP and academic achievement adjusted for either birth-related (Lambe et al., 2006) or familial characteristics (O'Callaghan et al., 2010), thus limiting the impact of potential confounders; however, academic achievement was not assessed at different time points. Consequently, the present study sought to employ a longitudinal assessment that would identify possible changes in the association between MSDP and academic achievement over time. In contrast, this study conflicts with studies by Gilman et al. (2008) and D'Onofrio et al. (2010); their results showed no association with MSDP and scholastic achievement. A review from Clifford et al. (2012) also identified similar null relationships, or slight relationships, which were later attenuated by other factors.

This study has several noteworthy strengths. First, we employed a longitudinal cohort design that permitted assessments at three time points. To our knowledge, this is the first study to employ such a design in assessing the relationship between MSDP and academic achievement. Second, the use of registry data is more likely to circumvent random errors associated with self-reports and thus strengthens the validity of our findings. Third, the use of an analytic approach that accounts for the correlation of data adds robustness to the methodology and findings. These strengths notwithstanding, this study is not without limitations. First, no data were available with which we could verify the smoking status of mothers or determine the duration or frequency of smoking beyond the first trimester of pregnancy. Second, we were not able to capture data regarding exposure to second-hand smoking from, for example, a smoking partner or other family member. Moreover, neurodevelopmental outcomes may be partly influenced by genes and maternal education (Huijbregts et al., 2006; Skoglund et al., 2014); however, we were unable to obtain data on parental education levels or on genetic inheritance. Finally, maternal IQ, quality of the home environment, mother-child relationship, parental roles and performance, and the number of siblings could not be assessed.

## 5 Conclusions

Maternal smoking during pregnancy exerts a negative impact on academic achievement beginning in childhood and going well into early adolescence. These negative effects persist irrespective of gender and time. Smoking during the first trimester of pregnancy may have long-lasting detrimental impact on the academic potential of offspring. Future research should seek to replicate these findings among more diverse populations, while controlling for maternal education levels, and cumulative smoking exposure throughout the entire pregnancy.

## Funding

This work was supported by the European Research Council (ERC-CoG-2014-647860).

## Declaration of interests

Conflict of interest: none declared.
